# Antihyperlipidemic and Antioxidative Potentials of Onion (*Allium cepa *L.) Extract Fermented with a Novel* Lactobacillus casei* HD-010

**DOI:** 10.1155/2019/3269047

**Published:** 2019-03-03

**Authors:** Woong-Suk Yang, Jin-Chul Kim, Jae Yong Lee, Cheorl-Ho Kim, Cher-Won Hwang

**Affiliations:** ^1^Nodaji Co., Ltd., Pohang, Gyeongbuk, Republic of Korea; ^2^School of Life Sciences and Biotechnology, Kyungpook National University, Daegu, Republic of Korea; ^3^Natural Constituents Research Center, Natural Products Research Institute, Korea Institute of Science and Technology, Gangneung, Republic of Korea; ^4^Department of Advanced Aerospace Materials Egineering, Kyungwoon University, Gumi, Gyeongbuk, Republic of Korea; ^5^Department of Biological Sciences, College of Science, SungKyunKwan University, Seoburo 2066, Jangan-Gu, Suwon 16419, Republic of Korea; ^6^Department of AGEE, Handong University, Pohang, Gyeongbuk, Republic of Korea

## Abstract

The purpose of this study was to investigate antihyperlipidemic and antioxidative potentials of onion (*Allium cepa* L.) extract fermented with a novel* Lactobacillus casei* HD-010. In general, fermented onion extract is used for its antioxidative activity (ORAC), inhibitory effect on adipocytes differentiation, quercetin contents, and antihyperlipidemic activities. However, the effect of fermented onion extract on hyperlipidemia after oral administration using ApoE-deficient mice has not been reported yet. To understand the effect of fermented onion extract on hyperlipidemia, we used benzafibrate (10 mg/kg, bw/day) as a positive control in the present study. Serum was collected every week to analyze levels of low density lipoprotein (LDL), high density lipoprotein (HDL), triglyceride (TG), and cholesterol, 3-hydroxy-3-methylgutaryi-CoA (HMG-CoA) reductase activity, and cholesterol ester transport protein (CETP) activity. In the fermented onion-treated group, HDL level was significantly increased while levels of TG and LDL were significantly decreased compared to those in the control group. In addition, the inhibition activity of HMG-CoA reductase was increased 20% in the fermented onion-treated group at 100 mg/kg. CETP activity has been observed to be significantly inhibited in the fermented onion-treated groups compared to that in the control group. These results suggest that fermented onion has a preventive/therapeutic effect on hyperlipidemic disease. It might have potential to be developed as a functional food.

## 1. Introduction

Recently, food consumption pattern has considerably changed from traditional fermented food-based intake (Kimchi, fermented soy bean, etc.) to fat containing westernized diet (meat, fats, etc.) in Asia, including Korea [1–3]. Westernized food intake pattern is known to increase risks of obesity, high blood pressure, diabetes, and hyperlipidemia [4–7]. Hyperlipidemia is a risk factor of cardiovascular diseases. Controlling hypercholesterolemia is important to prevent hyperlipidemia. Reducing triglyceride level in blood stream is one of treatments for patients with cardiovascular related disease through inducing LDL receptors and limiting VLDL secretion with certain drugs [8].

There are several medicines for reducing hyperlipidemia symptoms, such as HMG-CoA reductase inhibitor (statins), PPAR-alpha activator (fibrate), CETP inhibitor, bile acid sequestrants, and ACAT inhibitor [9–11]. However, long-term treatment with these medications has side effects. Thus, many studies have tried to increase drug efficiency [12, 13].

Reducing cholesterol concentration in blood is an important research issue for functional food and drug development to decrease the risk of cardiovascular related diseases. Natural components from plant or organisms are potential candidates to decrease the risk of disease outbreak. Onion (*Allium cepa* L.) has been used for decreasing blood cholesterol levels [14]. In Asia, it was traditionally used as a medicine due to its fever-reducing, antiparasitic, detoxification, and intestinal anti-inflammation effects [5, 15–17]. Major compounds in onion are flavonoids (quercetin, quercitrin, and rutin) and sulfuric compounds (allyl propyl disulfide, diallyl disulfide) with health improving effects [18]. Another method to reduce cholesterol is by using* Lactobacillus* for fermentation.* Lactobacillus* has been studied for its cholesterol-reducing effect. Klaver et al. [19] have reported that* Lactobacillus* can deconjugate bile acid and inhibit the function of cholesterol. However, the effect of fermented onion extract on hyperlipidemia after oral administration using ApoE-deficient mice has not been reported yet. Therefore, the purpose of this study was focused on antihyperlipidemic and antioxidative potentials of fermented onion (*Allium cepa* L.) with a novel* Lactobacillus casei* HD-010 in lipid metabolism.

## 2. Materials and Methods

### 2.1. Selection of Bacterial Strain and Culture Condition

Ten strains were identified from fermented onion and the main strain was* Lactobacillus casei *HD-010 (Table 1). We used* L. casei* KCTC 2180 from Korean Collection for Type Cultures as a positive control. The identified strain* L. casei* HD-010 was cultured at 30°C for 10 days to ferment onion extract. Onion extract was prepared with minced clean onion after washing with double distilled water three times. Autoclaved onion extract at 121°C for 15 minutes was used for fermentation. Strain identification medium was prepared using 5.5% MRS broth (Difco, France) with 2.0% agar (Difco, France). Liquid culture media was prepared as the strain identification media without 2.0% agar.

### 2.2. Preparation of Fermented Onion Extract

A 30-liter fermenter (Biostat C Plus, Sartorius, Sweden) [20, 21] was used for onion extract fermentation with 100% onion extract under sterile condition. After cooling the onion extract, 1% HD-010 which was incubated at 37°C with shaking (200 rpm) for 24 hours inoculated into the fermenter and cultured at 37°C with shaking (25 rpm) for 10 days. After filtering the fermented onion extract with a filter (0.2 *μ*m pore size), the extract was lyophilized (PVTFD20RS, Ilshin Lab. Co. Ltd., Korea) and kept at -80°C until the experiment was performed. As a positive control,* L. casei *KCTC 2180 was used. It was prepared with the same method as* L. casei *HD-010.

### 2.3. Assay of Oxygen Radical Absorbance Capacity (ORAC)

Antioxidant capacities of fermented onions, layers of organic solvents, fractions, and subfractions were determined using ORAC assay as described by Gillespie et al. [22]. Briefly, samples or Trolox (0, 6.25, 12.5, 25, 50, and 100 *μ*g/ml) were mixed with phosphate buffered saline (75 mmol/L, pH 7.4, Thermofisher scientific, Waltham, MA, USA). After the addition of *β*-phycoerythrin (0.2 mmol/L) and 2,2'-azobis(2-amidinopropane) dihydrochloride (AAPH, 200 mmol/L, Wako Pure Chemical Industries, Ltd., Osaka, Japan) as radical generators were added to wells of a 96-well plate. Fluorescence was measured with a fluorescence ELISA reader (VICTOR®, PerkinElmer, USA) every two minutes for sixty minutes (excitation wavelength: 535 nm, emission wavelength: 590 nm). The equation used to obtain AUC (area under the curve) was as follows:

(1) AUC=1+f1f0+f2f0+f3f0+…+f59f0+f60f0

where* f0 *was the initial fluorescence reading at 0 min and* fi *was the fluorescence reading at* i* (from 1 to 60) minutes.

(2) Relative ORAC value=AUCsample−AUCblankAUCTrolox−AUCblank×Molarity of TroloxMolarity of sample

### 2.4. Adipocyte Cell Culture and Differentiation

We purchased 3T3-L1 cell lines from the American Type Culture Collection (ATCC, USA). 3T3-L1 preadipocytes cells were plating into 96-well plates at a density of 1 × 10^4^ cells per well. And cultured at 37°C with 5% CO_2_ in Dulbeco's Modified Eagle Media (DMEM, Gibco, Invitrogen, USA) medium supplemented with 10% newborn calf serum (Gibco, Invitrogen, USA) and 100 U/ml penicillin-streptomycin (Gibco, Invitrogen, USA). Next, 3T3-L1 preadipocytes cells were cultured in differentiation medium (MDI) containing 10% fetal bovine serum (FBS, Gibco), 10 *μ*g/ml insulin (Sigma-Aldrich), 0.5 mM 3-isobutyl-1-methylxanthine (IBMX, Sigma-Aldrich), and 1 *μ*M dexamethasone (Sigma-Aldrich). Two days after stimulation with differentiation inducer (MDI, including 0.5 mM IBMX, 1 *μ*M dexamethasone, and 10 *μ*g/ml insulin), the medium was switched to DMEM containing 10% FBS and 10 *μ*g/ml insulin. Two days later, the medium was changed again to 10% FBS/DMEM. Cells were cultured in 10% FBS/DMEM every two days. Full differentiation was achieved by day 8. Onion extract samples were added to 3T3-L1 cells culture at various concentrations (6.25 ~ 100 *μ*g/ml) on four days after differentiation induction.

Intracellular lipid content was measured in 96-well plates using AdipoRed™ assay reagent (Cambrex, MA, USA). On day 8, the treatment medium was removed and the cells were fixed in a 4% formaldehyde solution at room temperature (25°C) for 5 hrs. After rinsing cells with PBS, each well was added with 200 *μ*l PBS and 5 *μ*l of AdipoRed reagent. Following incubation at room temperature for 10 min, plates were measured with a fluorescence ELISA reader (VICTOR®, PerkinElmer, USA) at an excitation wavelength of 485 nm and an emission wavelength of 535 nm. Values from each group were used to calculate the 50% effective inhibition concentration (EC_50_) for reducing adipocyte differentiation. As positive controls, benzafibrate and simvastatin were used.

### 2.5. Separation and Fractionation of Onion Extract Fermented with* L. casei* HD-010

Freeze-dried fermented onion extracts were resuspended in distilled water and partitioned with four different organic solvents (*n*-hexane, CH_2_Cl_2_, ethyl acetate, and* n*-butanol) and residual H_2_O. These fractions were subjected to decompression enriching and freeze-drying to remove residual solvent. The CH_2_Cl_2_ layers were sequentially applied to HP-20, silica gel and RP-C_18_ open column chromatography under the same column conditions (3.8 x 60 cm, 300 g) [23–25] to obtain the active compound from fermented onions with* L. casei* HD-010 (LFAc).

### 2.6. Quercetin Contents

Quercetin contents in fermented onion extracts were quantitatively analyzed [26] by analytical HPLC (Shimadzu CBM-20A Network LC system with LC-6AD pump, SPD-M20APDA detector, equipped with an SIL-10AF series automated liquid sampler). An Eclipse Plus-C_18_ column (Agilent, 3.0 x 100 mm, 0.35 *μ*m) was used at the following conditions: flow rate, 1.0 ml/min; total run length, 30 minutes; mobile phase 90% ACN plus 0.02 M KH_2_PO_4_(pH 2.0 with H_3_PO_4_); injection volume of samples or STD, 20 *μ*l; and wavelength, 372 nm. Quercetin (Q4951) was used as comparative derivative standard (CAS No. 117-39-5, Sigma-Aldrich, USA)

### 2.7. Animal Experiments

Male ApoE-deficient mice (five weeks old) were supplied from Central Laboratory Animal Inc. in Korea and housed at 23 ± 0.5°C with 55 ± 7% humidity and light-dark cycle (12 hrs*  *: 12 hrs). All animals were acclimated at least one week. They were caged and fed a low fat, low cholesterol control diet D12336 (Central Laboratory Animal Inc., Seoul, Korea).

All animal studies were performed in a pathogen-free barrier zone at Kyungpook National University. All procedures used in this study were approved by the Animal Care and Use Committee of Kyungpook National University (IACUC approval number: KNU2012-136).

**Figure 1:**
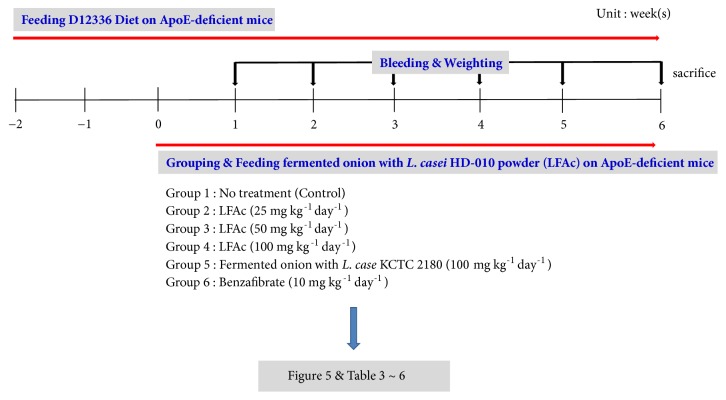
Animal experiment design.

The control group was fed with a high fat diet. The positive control group was fed with benzafibrate (10 mg/kg). Fermented onion extract was fed into three groups with different amounts by oral administration in 0.5 ml of saline (low dose, 25 mg/kg; medium dose, 50 mg/kg; and high dose, 100 mg/kg). Saline alone group was used as a negative control (N=10/group) [24, 27]. Animal experiment design of this study is shown in Figure 1.

#### 2.7.1. Lipid Contents Measurement

Blood was collected from mouse by retroorbital sinus bleeding method using intraorbital venous plexus every week for six weeks. Blood samples were incubated at room temperature for 30 minutes and centrifuged at 600 g for 10 minutes at 4°C. Sera samples were prepared and kept at -80°C until assay. HMG-CoA reductase and CETP inhibition activities were measured using sera samples collected at the last experimental point (6^th^ week samples). HMG-CoA reductase and CETP inhibition activities were measured using HMG-CoA reductase assay kit (Sigma, USA) and CETP assay kit (Biovision, USA), respectively. Serum was measured for contents of total cholesterol (TC), LDL-cholesterol (LDL-C), HDL-cholesterol (HDL-C), triglyceride (TG) using Asan kit (Asan medical company, Korea) and a Beckman Coulter biochemical analyzer.

### 2.8. Statistical Analysis

The results are presented as mean ± standard deviation (mean ± SD). The statistical analyses of data were determined by using two-tailed Student's* t-*test.

## 3. Results and Discussion

### 3.1. Fermented Onion Exhibits Antioxidative Activity

We have investigated the anti-oxidative activity of onion extract fermented with* L. casei* HD-010 (LFAc) by ORAC assay. LFAc had higher ORAC value than Trolox, a positive control (ORAC_PE_ of LFAc extract = 1.02).

**Figure 2:**
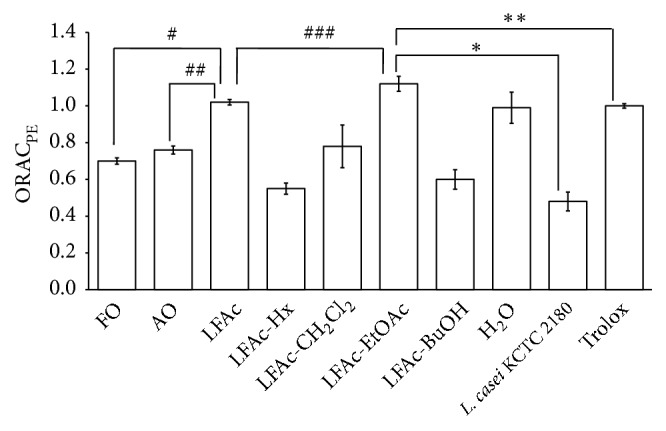
Antioxidative activities of fractions of onion extract fermented with* L. casei*HD-010 (LFAc) using organic solvents. Oxygen Radical Absorbance Capacity (ORACPE) values were obtained in the antioxidant assay using various organic solvent fractions. Trolox was used as a positive control (ORAC_PE_ value was 1.00). Data are presented as mean ± SD (n = 3). #*p*<0.05 versus control group (FO treated group); ##*p*<0.05 versus control group (AO treated group); ###*p*<0.05 versus LFAc treated group; *∗p*<0.05 versus positive control group (*L. casei* KCTC 2180 treated group); *∗∗p*<0.05 versus positive control (Trolox treated group); FO, fresh onion; AO, autoclave onion; LFAc, fermented onion with* L. casei* HD-010; Hx,* n*-hexane; CH_2_Cl_2_, dichloromethane; EtOAc, ethyl acetate; BuOH,* n*-butanol;* L. casei* KCTC 2180, fermented onion with* L. casei* KCTC 2180.

In order to determine which fractions of the onion extracts fermented with* L. casei* HD-010 contained antioxidative ingredients, we further separated the extract using four different organic solvents as described in the Materials and Methods section. The LFAc-EtOAc fractions had the highest ORAC value (ORAC_PE_ of LFAc-EtOAc = 1.12) (Figure 2), suggesting that the EtOAc fractions of the onion extract fermented with* L. casei* HD-010 (LFAc) contained an antioxidative component. This result suggests that onion extract fermented with* L, casei* HD-010 (LFAc) has antioxidative activity.

### 3.2. Adipocyte Differentiation Inhibition

**Figure 3:**
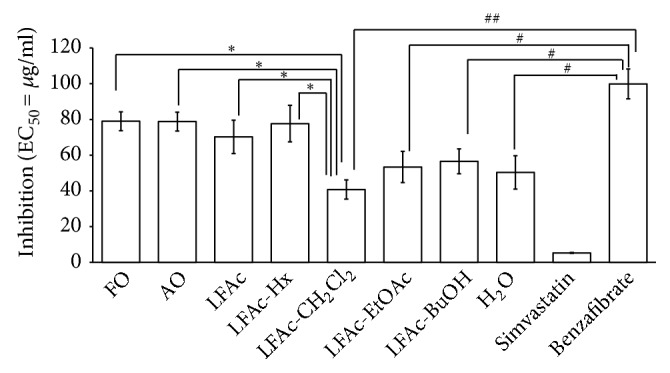
Adipocyte differentiation inhibition of onion (*Allium cepa *L.) extract fermented with* L. casei *HD-010 (LFAc). Data are presented as mean ± SD (n = 3). *∗p*<0.05 versus FO, AO, LFAc, or LFAc-Hx treated group; #*p*<0.05 versus positive control (benzafibrate treated group); ##*p*<0.01 versus positive control (benzafibrate treated group). FO, fresh onion; AO, autoclave onion; LFAc, fermented onion with* L. casei *HD-010; Hx,* n*-hexane; CH_2_Cl_2_, dichloromethane; EtOAc, ethyl acetate; BuOH,* n*-butanol.

Fermented onion with* L. casei* HD-010 (LFAc) showed an inhibitory effect on adipocytes differentiation compared to fresh onion or autoclaved onion (> 20%). The inhibitory effect of LFAc was specifically observed in the CH_2_Cl_2_ layer (> 45%) (Figure 3). As a positive control, benzafibrate had no effect on differentiation. However, simvastatin treatment showed more than 90% of differentiation inhibition. Therefore, LFAc has an inhibitory function by blocking HMG-CoA reductase activity.

### 3.3. Dichloromethane Layers of Onion Extract Fermented with* L. casei* HD-010 (LFAc) Have Both Adipocytes Differentiation Inhibiting and Antioxidative Effects

In order to purify and identify the active compound in LFAc for the induction of physiological activity, CH_2_Cl_2_layers were subjected to several isolation procedures (HP-20, Silica gel, and RP-C_18_ open column). HLFAc-30 and SLFAc-4 fractions with strong adipocyte differentiation inhibiting activities were sequentially obtained (date not shown) after further isolation of SLFAc-4 fractions in an RP-C_18_ open column.

In order to investigate hyperlipidemia inhibitory function after LFAc, MC fraction was subjected to HP-20, silica gel, and RP-18 open chromatography. Antioxidant and adipocytes differentiation inhibition effects were observed for LFAc-HP3 fraction from HP-20, LFAc-S4 fraction from silica gel, and LFAc-C3 from C1 (Table 2).

### 3.4. Quercetin Contents

**Figure 4:**
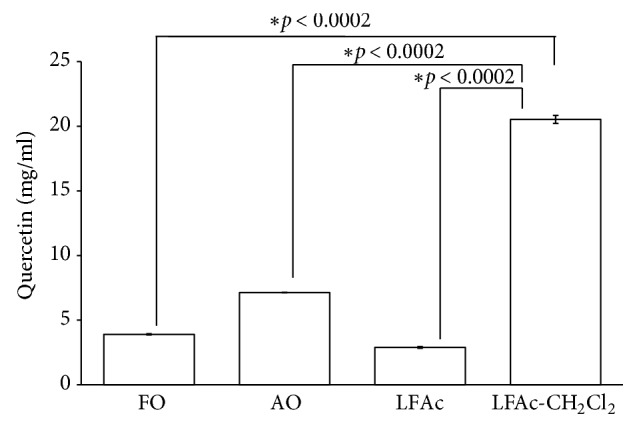
Quantity of quercetin contents in onion (*Allium cepa *L.) extract with or without fermentation using HPLC analysis. Data are presented as mean ± SD (n = 3). FO, fresh onion; AO, autoclave onion; LFAc, fermented onion with* L. casei *HD-010; CH_2_Cl_2_, dichloromethane.

Thin layer chromatography (TLC) was used to separate components from raw onion extract (FO), sterilized onion extract (AO), and fermented onion extract (LFAc). The pattern was not different between samples and four major spots were found (data not shown). LFAc-C4 fraction showed the best adipocytes differentiation inhibitory effect and an effective single fraction was identified. Quercetin, one of major onion components, was identified from LFAc-C4 fraction. FO, AO, LFAc, and LFAc_CH_2_Cl_2_ were examined with HPLC. Quercetin contents in these fractions were FO, 3.90 ± 0.041 mg/ml; AO, 7.13 ± 0.009 mg/ml; LFAc, 2.89 ± 0.064 mg/ml; and LFAc_CH_2_Cl_2_, 20.53 ± 0.304 mg/ml. Quercetin content was not altered by fermentation procedure. However, following fermentation with probiotics, quercetin content was enhanced by almost 10-fold in LFAc-CH_2_Cl_2_ (Figure 4).

### 3.5. Animal Test

#### 3.5.1. Body Weight

The effect of fermented onion extract on body weight was tested for six weeks using mice with a high fat diet. Any significant decrease in body weight has been observed in the fermented onion extract feeding group. Dietary fiber, flavonoids, and sulfuric components in onion efficiently reduced their body weights compared to the high fat diet alone group (data not shown). This result suggests that oral administration of fermented onion extract has no direct effect on body weight, consistent with other studies [18].

#### 3.5.2. Serum Lipid Contents Measurement

Serum was collected every week for six weeks and assessed for changes in LDL-C, HDL-C, TG, and TC contents. At the end of the experiment, serum was tested for HMG-CoA reductase and CETP inhibition effect. Fermented onion extract feeding groups (low, medium, and high) showed significant decreases in LDL-C level from the fifth week. The medium and high fermented onion extract feeding groups showed continuous decrease in their body weights (Table 3). In addition, HDL-C level was increased from the first week until the sixth week after administration (Table 4). The LSP-11 supernatant feeding group showed considerable changes in HDL-C and LDL-C levels at the third and fifth weeks. These data suggest that fermented onion extract might have synergic effects on functions of secondary metabolites from* Lactobacillus casei *HD-010.

Serum TG level was slightly decreased in all groups compared to that in the control. However, such decrease was not statistically significant. Specifically, high fermented onion extract fed group showed significant decrease in TG level at the first, second, third, and fifth week (Table 5). TC level was reduced in the fermented onion extract fed group from the fifth week (Table 6). However, the positive control group that was fed with benzafibrate and* Lactobacillus* supernatant did not show any significant difference in TC level, when compared to the control group.

**Figure 5:**
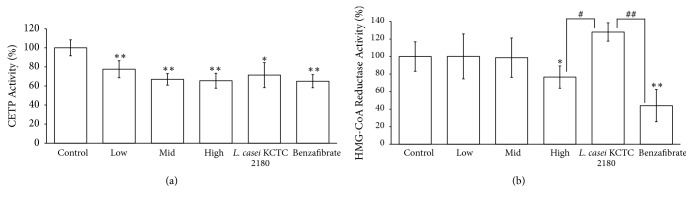
Effect of fermented onion with* L. casei*HD-010 on serum CETP and HMG- CoA reductase activity in ApoE-deficient mice at six weeks. Data are presented as mean ± SD (10 animals per group; three independent experiments were performed). Statistical significance between untreated and treated values was determined by two-tailed Student's* t-*test and is given as a* p *value; *∗p *value < 0.05 and *∗∗p *value < 0.001(versus control group); #*p *value < 0.05 and ##*p *value < 0.001 (versus* L. casei* KCTC 2180 and benzafibrate treated group).

We used ApoE-deficient mice model in order to assess the efficiency of fermented onion extract on reducing lipid accumulation, HMG-CoA reductase inhibition, and CETP inhibition. HMG-CoA reductase is involved in cholesterol synthesis [13, 28]. It was reduced following fermented onion extract administration. However, such decrease was not statistically significant (Figure 5). CETP protein works as a carrier for HDL and LDL into the body [3, 29] and HMG-CoA reductase is involved in cholesterol synthesis [13, 28]. As shown in Figure 5, CETP activity and HMG-CoA reductase were significantly following fermented onion extract administration (Figure 5). These data suggest that fermented onion extract can efficiently block intestinal fat adsorption through inhibition of CETP activity.

Hyperlipidemia is an important issue in healthcare. It is involved in many serious cardiovascular diseases. Many experimental and clinical studies have shown that hyperlipidemia may cause hypertension, diabetes, and obesity [4, 6, 7, 19].

Many studies have reported that onion components or* Lactobacillus* can lower lipid contents in blood. Onion is a well-known traditional medicine. It has been investigated for many epidemiologic studies [19, 30]. In Asian countries, onion and garlic plants that contain diallyl sulfide and quercetin are used to prevent cardiovascular diseases. Onion contains about 90% water, 7 ~8% sugar (mainly fructose), and minor amounts of vitamins [29, 31]. S-methyl-L-cysteine sulphoxide is one of the components in onion. It can reduce lipid contents in blood [14, 16]. Quercetin has a similar effect in reducing lipid production and synthesis in animal experiment [32].* Lactobacillus* can also reduce cholesterol level in blood. Many studies have shown that* Lactobacillus* can inhibit bile acid readsorption and attachment of cholesterol onto cell wall [33, 34]. However, fermented onion extract has not yet been studied well. Few research groups have attempted to develop fermented onion extract drink product.

In this study, we attempted to identify a proper bacterium for onion fermentation and determine its effect on blood lipid level. Our data suggest that fermented onion extract has an effect on lipid metabolism by oral administration.

## 4. Conclusions

The main active material responsible for the antihyperlipidemia effect of fermented onion was quercetin. Our results suggest that fermented onion has preventive/therapeutic effect on hyperlipidemic disease. It might have potential to be developed as a functional food.

## Figures and Tables

**Table 1:** Identification of isolated bacteria by 16s-rRNA.

Code Name	Results	Homology (%)
HD-001	*Bradyrhizobium japonicum*	97
HD-002	*Bacillus *sp.	95
HD-003	*Bacillus *sp.	95
HD-004	*Bacillus clausii*	89
HD-005	*Janibacter *sp.	96
HD-006	*Bacillus clausii*	90
HD-007	*Burkholderia tropica*	100
HD-008	*Bacillus *sp.	97
HD-009	*Paenibacillus *sp.	100
HD-010	*Lactobacillus casei*	100

[Ref. https://blast.ncbi.nlm.nih.gov/Blast.cgi]

**Table 2:** Adipocyte differentiation inhibition and antioxidative activities of subfractions from LFAc_S4 using C_18_ open column.

Samples	Inhibition (EC_50_ = *μ*g/ml)	ORAC_PE_
LFAc_CH_2_Cl_2_	53.25	1.10 ± 0.015*∗∗*
LFAc_C1	53.41	1.06 ± 0.028
LFAc_C2	56.56	1.04 ± 0.013
LFAc_C3	40.25*∗*	1.15 ± 0.021*∗∗*
LFAc_C4	42.98*∗*	1.16 ± 0.057*∗∗*
LFAc_C5	>100	ND
Trolox	ND	1.00 ± 0.017

Data are presented as mean ± SD (n = 3); *∗p*<0.05 versus control group (PBS treated group); *∗∗p*<0.05 versuspositive control (Trolox treated group). ND (not detected).

**Table 3:** Effect of fermented onion with *L. casei* HD-010 on serum low-density lipoprotein level in ApoE-deficient mice.

Treatment	Dose (mg/kg/day)	Low density lipoprotein (LDL, mg/dl)						
		0 weeks	1 week	2 weeks	3 weeks	4 weeks	5 weeks	6 weeks
Control	0	575.9±51.05	620.0±96.49	536.6±93.56	621.9±47.44	509.8±67.23	675.9±54.93	592.4±37.39
Low	25	581.4±81.00	624.1±58.78	462.3±72.85^#^	624.4±26.62	484.6±52.17^#^	517.0±92.00^*∗*^	553.5±40.53
Mid	50	532.6±81.58	605.1±63.79	441.3±72.70^#^	597.1±55.23^#^	477.1±98.76^#^	486.7±59.18^*∗∗*##^	547.4±31.61^*∗*^
High	100	517.0±39.48	595.9±64.42	336.4±62.60^*∗∗*##^	591.0±89.04^##^	454.9±20.30^##^	484.2±69.66^*∗∗*##^	500.3±77.92^*∗*#^
*L. casei *KCTC 2180	100	612.6±60.79	677.9±114.79	593.6±47.41	723.1±28.63^*∗∗*^	625.6±55.93^*∗*^	685.7±72.79	624.0±26.55
Benzafibrate	10	597.3±90.47	581.8±40.11	513.4±67.09	652.3±83.81	649.9±69.99^*∗*^	652.4±76.33	590.0±24.63

Data are presented as mean ± SD (10 animals per group; three independent experiments were performed).

Statistical significance between control and treated values was determined by two-tailed Student's *t-*testwith *p *value; *∗p *value < 0.05 and *∗∗p *value < 0.001 (versus control group); #*p *value < 0.05 and ##*p *value < 0.001 (versus *L. casei* KCTC 2180 and benzafibrate treated group).

**Table 4:** Effect of fermented onion with *L. casei* HD-010 on serum high-density lipoprotein level in ApoE-deficient mice.

Treatment	Dose (mg/kg/day)	High density lipoprotein (HDL, mg/ dl)						
		0 weeks	1 week	2 weeks	3 weeks	4 weeks	5 weeks	6 weeks
Control	0	45.5±7.41	47.1±1.05	44.3±8.95	50.8±3.20	36.0±4.23	31.4±4.43	44.8±2.27
Low	25	45.5±1.06	50.7±1.91^*∗∗*^	46.2±7.38	57.4±1.28^*∗∗*^	56.6±9.70^*∗∗*^	48.0±9.51^*∗∗*^	45.6±8.59
Mid	50	52.8±3.02	58.2±3.77^*∗∗*^	52.9±6.82^##^	65.3±0.92^*∗∗*^	58.6±4.10^*∗∗*^	51.8±3.41^*∗∗*^	56.2±7.73^*∗∗*#^
High	100	56.6±8.96	64.2±6.00^*∗∗*#^	55.4±6.81^##^	70.3±3.64^*∗∗*##^	66.3±4.18^*∗∗*##^	62.5±5.13^*∗∗*##^	56.6±1.98^*∗*#*∗*^
*L. casei *KCTC 2180	100	45.2±3.90	52.7±3.95^*∗*^	38.9±5.06	54.4±2.40	46.3±6.13^*∗*^	48.8±8.62^*∗∗*^	43.6±6.55
Benzafibrate	10	43.2±2.95	48.0±2.99	39.1±5.29	55.7±4.66	35.8±0.92^*∗∗*^	32.1±5.85	42.7±6.30

Data are presented as mean ± SD (10 animals per group; three independent experiments were performed).

Statistical significance between control and treated values was determined by two-tailed Student's *t-*testand is given as a *p *value; *∗p *value < 0.05 and *∗∗p *value < 0.001(versus control group); #*p *value < 0.05 and ##*p *value < 0.001 (versus * L. casei* KCTC 2180 and benzafibrate treated group).

**Table 5:** Effect of fermented onion with *L. casei* HD-010 on serum triglyceride level in ApoE-deficient mice.

Treatment	Dose (mg/kg/day)	Triglyceride (TG, mg/ dl)						
		0 weeks	1 week	2 weeks	3 weeks	4 weeks	5 weeks	6 weeks
Control	0	406.6±35.68	561.2±54.01	334.4±60.37	652.3±65.16	302.3±48.56	412.2±75.99	284.8±54.11
Low	25	379.1±76.36	553.5±70.59	301.0±75.14	628.2±63.90	288.3±45.88	396.2±59.68	266.6±23.08
Mid	50	363.5±64.19	461.6±99.73	270.1±16.91	581.1±34.41	283.1±43.47	321.0±82.27	265.7±16.24
High	100	395.5±61.04	449.7±59.96^*∗*^	228.5±42.10^*∗*^	537.3±62.24^*∗*^	273.4±60.84	241.5±68.03^*∗∗*^	252.5±39.91
*L. casei *KCTC 2180	100	410.4±78.77	475.9±41.84^*∗*^	275.2±37.67	507.0±24.04^*∗∗*^	241.7±57.09	255.9±65.39^*∗∗*^	270.3±26.89
Benzafibrate	10	380.5±86.76	529.5±90.37	285.3±52.13	656.7±71.61	257.2±33.35	396.9±99.12	273.6±39.73

Data are presented as mean ± SD (10 animals per group; three independent experiments were performed).

Statistical significance between control and treated values was determined by two-tailed Student's *t-*testand is given as a *p *value; *∗p *value < 0.05 and *∗∗p *value < 0.001.

**Table 6:** Effect of fermented onion with *L. casei* HD-010 on serum total cholesterol level in ApoE-deficient mice.

Treatment	Dose (mg/kg/day)	Total Cholesterol (TC, mg/dl)						
		0 weeks	1 week	2 weeks	3 weeks	4 weeks	5 weeks	6 weeks
Control	0	702.7±40.72	779.4±88.99	647.8±89.39	803.2±41.15	621.8±71.15	789.7±55.05	699.1±41.20
Low	25	702.7±88.48	785.5±63.45	568.7±82.61	807.5±19.50	598.8±46.83	644.3±79.84^*∗*^	644.8±39.13^*∗*^
Mid	50	658.1±73.41	755.7±51.34	548.3±67.92	778.6±57.04	592.3±91.15	602.7±62.89^*∗∗*^	641.1±60.00^*∗*^
High	100	652.7±28.06	750.1±60.31	437.4±61.70^*∗∗*##^	768.7±83.53^#^	575.9±20.40^#^	595.0±73.14^*∗∗*#^	619.4±78.28^*∗*^
*L. casei* KCTC 2180	100	739.9±48.37	825.8±113.75	687.6±52.21	879.0±32.08^*∗*^	720.2±66.89	785.8±79.01	715.2±33.01
Benzafibrate	10	716.6±90.81	735.7±26.94	609.5±66.18	839.3±92.52	737.1±73.97^*∗*^	763.9±87.42	687.4±19.36

Data represent mean ± SD(10 animals per group; three independent experiments were performed).

Statistical significance between control and treated values was determined by two-tailed Student's *t-*testand is given as a *p *value; *∗p *value < 0.05 and *∗∗p *value < 0.001(versus control group); #*p *value < 0.05 and ##*p *value < 0.001 (versus * L. casei* KCTC 2180 and benzafibrate treated group).

## Data Availability

The data are linked to online repositories of http://www.nodagi.net.
